# The Role of Medial Prefrontal Cortex in Acupuncture Treatment for Functional Dyspepsia

**DOI:** 10.3389/fnins.2022.801899

**Published:** 2022-04-07

**Authors:** Yuke Teng, Tao Yin, Yue Yang, Ruirui Sun, Zilei Tian, Peihong Ma, Zhaoxuan He, Yuzhu Qu, Liuyang Huang, Yuan Chen, Fang Zeng

**Affiliations:** ^1^Acupuncture and Tuina School/The 3rd Teaching Hospital, Chengdu University of Traditional Chinese Medicine, Chengdu, China; ^2^School of Acupuncture-Moxibustion and Tuina, Beijing University of Chinese Medicine, Beijing, China; ^3^International Education School, Chengdu University of Traditional Chinese Medicine, Chengdu, China

**Keywords:** acupuncture, functional dyspepsia, curative effect, fMRI, mPFC, DMN

## Abstract

Acupuncture is an effective therapy for functional dyspepsia (FD). However, the efficacy of acupuncture in the treatment of FD varies among individuals in clinical practice. This study aimed to reveal the brain response patterns in acupuncture higher response/lower response FD patients. Firstly, we performed a within-group comparison of brain function activity before and after acupuncture treatment in 115 FD patients and analyzed the correlation between brain function activity changes and clinical improvements. Secondly, 115 subjects were divided into the acupuncture higher response group or the lower response group based on the median clinical improvement values. The changes in functional brain activity after acupuncture treatment were investigated in these two groups, respectively. Finally, the identified brain regions associated with the clinical improvements were set as regions of interest (ROI), and the ROI-to-voxel functional connectivity comparisons were also performed in both groups, respectively. The results demonstrated that the functional activities of the left cerebellum inferior, right middle temporal gyrus, and right medial prefrontal cortex (mPFC) were increased, and the left Heschl and right middle cingulate cortex were decreased in 115 FD patients after acupuncture treatment. The functional connectivity changes of mPFC were correlated with improving the Nepean Dyspepsia Symptom Index. The significant increase in mPFC functional activity was also found in acupuncture higher response FD patients but not in lower response FD patients. The functional connectivity between the mPFC and default mode network (DMN) was significantly diminished in the higher response group but not in the lower response group. In conclusion, this study suggested that modulating the functional activity of the mPFC and its connectivity to the DMN may be one of the important mechanisms of acupuncture for treating FD with a higher response.

## Introduction

As a functional gastrointestinal disorder (FGID) with high prevalence in the general population (Enck et al., 2017), functional dyspepsia (FD) is characterized by postprandial fullness, early satiation, epigastric pain, or burning (Ford et al., 2020). FD significantly affects the quality of life (QoL) of patients and families, results in severe medication overuse, and leads to substantial social and financial burdens (Black et al., 2020; Oh et al., 2020). However, due to its complex etiology and unclear pathogenesis, the curative effect of FD is unsatisfactory (Ford et al., 2020), so efforts in seeking effective alternative therapies attract both patients and practitioners.

Acupuncture has been used to treat gastrointestinal symptoms for several millennia in China and some other Asian countries. It is increasingly accepted as an alternative treatment for FGIDs in western countries (Wang et al., 2021). In clinical practice, the efficacy of acupuncture in the treatment of FD varies among individuals (Yang et al., 2020). The varying effectiveness may be related to the different responses to acupuncture treatment. Therefore, it is necessary to explore the mechanism of the acupuncture effect to improve clinical efficacy.

The brain–gut axis is a conception of nerve anatomy. The central nervous system and myenteric nervous plexuses have a bidirectional connection that affects functions such as sensation and movement. With the development of neuroimaging technology, the study of the brain–gut axis is not limited to animal experiments. Non-invasive and high spatial and temporal resolution techniques, such as functional magnetic resonance imaging (fMRI), support brain–gut interaction studies based on human beings. For example, through fMRI techniques, studies have identified changes in brain activity in the medial prefrontal cortex, orbitofrontal cortex, cingulate gyrus, hippocampus, precuneus, and temporal pole in FD patients compared with healthy subjects (Liu et al., 2013; Chen et al., 2018; Qi et al., 2020). Thus, the Rome criteria have defined FD as a disease with abnormal brain–gut interaction (Drossman, 2016). In addition, these neuroimaging techniques have also been widely used to study the mechanisms of acupuncture treatment for FD. Multiple neuroimaging studies demonstrated that acupuncture treatment not only improved clinical symptoms (postprandial fullness, upper abdominal bloating, and early satiation) but also significantly modulated the abnormal brain function in FD patients, such as the posterior cingulate cortex, insula, and hippocampus, etc. (Zeng et al., 2015; Wang et al., 2020; Yang et al., 2020). However, whether the key to the therapeutic effects of acupuncture is related to the activity of some brain regions requires further research.

Therefore, based on the hypothesis that the brain response of acupuncture higher response FD patients is different from that of lower response patients, the present study aimed to (1) investigate the brain activity changes elicited by acupuncture treatment in all enrolled FD patients and analyze the correlation between symptom improvements and brain activity changes to explore the potential key regions involved in acupuncture treatment; (2) compare the differences in brain activity changes between the higher group (group exhibiting higher responses to acupuncture treatment) and the lower group (group exhibiting lower responses to acupuncture treatment) to verify further the key regions involved in acupuncture treatment; and (3) investigate the changes of connectivity pattern of the key regions in the higher response group, to explore the central mechanism of acupuncture for treating FD.

## Materials and Methods

This study is a secondary data analysis study. The data originate from two randomized controlled neuroimaging trials with acupuncture treatment for FD (The Chinese Clinical Trial Registry: ChiCTR-IOR-15006402 and ChiCTR-IOR-15006523) (Yin et al., 2017; Sun et al., 2018, 2021).

### Patients

A total of 115 FD patients from the two studies mentioned above were included in this study. All FD patients were from the outpatient department of the Affiliated Hospital of Chengdu University of Traditional Chinese Medicine and the campus of Chengdu University of Traditional Chinese Medicine. FD patients were enrolled if they fulfilled the following inclusion criteria: (1) they match the Rome III criteria for functional dyspepsia and postprandial distress syndrome, (2) they are right-handed and aged 18 to 45 years old, (3) they are not attending any other clinical trials in last 3 months, and (4) they provide written informed consent. Patients were excluded if they: (1) had esophagitis, gastric atrophy, or erosive gastroduodenal lesions on endoscopy; cholecystitis; and gallstones; (2) were pregnant or lactating; (3) were suffering from cardiovascular, renal, or respiratory illnesses or other organic diseases; (4) had a history of psychiatric and neurological disorders or head trauma with loss of consciousness; or (5) were using dynamic gastrointestinal medicine or received acupuncture treatment during the last 15 metal stents or electronic implant, intraocular metal foreign body, claustrophobia, hyperpyrexia, etc. (Yin et al., 2017; Sun et al., 2018).

### Acupuncture Interventions

The 115 FD patients received the 20 sessions’ acupuncture treatment over 4 weeks (five sessions per week). In each week, acupuncture was performed once per day for 5 days continuously, with 2-day intervals to the next week. In each session, patients received manual acupuncture treatment with disposable sterile stainless-steel needles (25–40 × 0.25 mm; Suzhou Hua Tuo Medical Instrument Co., Ltd., China) for 30 min. Acupuncture treatment was performed by two licensed acupuncturists with experience of over 3 years. In the two original studies, one study used acupuncture ST36 combined with CV12 as the treatment group and acupuncture ST36 and acupuncture CV12 as the control groups, respectively; the other study used acupuncture ST36 with Deqi as the treatment group and without Deqi as the control group, respectively (Yin et al., 2017; Sun et al., 2018).

### Outcome Measurements

The outcome measurements of the two studies included the Nepean Dyspepsia Symptom Index (NDSI) and Symptom Index of Dyspepsia (SID). The NDSI is a dyspepsia-specific index used to measure the dyspepsia symptoms over the previous 14 days. The scale measures the frequency, intensity, and level of discomfort of 15 upper gastrointestinal symptoms. It includes interference (13 items), knows/control (7 items), eats/drink (3 items), and sleep/disturb (2 items), and higher scores indicate more severe symptoms and poorer QoL. The SID includes the evaluation of four primary symptoms of FD, including postprandial fullness discomfort, early satiety, epigastric pain, and epigastric burning. The scoring of each item from none to severe is 0–3, with 0 indicating no symptoms, 1 indicating mild symptoms, 2 indicating moderate symptoms, and 3 indicating severe symptoms. The total score of four items evaluated the symptom severity of FD patients.

### fMRI Data Acquisition

All 115 FD patients received two fMRI scans, performed at baseline and after treatment. The parameters and sequences of the two fMRI scans were the same in all FD patients (Yin et al., 2017; Sun et al., 2018).

### Data Analysis

#### fMRI Data Processing

The fMRI data preprocessing was performed using SPM12 (SPM12^1^) and DPARSF 4.5^2^. The procedure was conducted as follows: (1) The first 10 time points of the fMRI scan for each subject were discarded, and the retained data were corrected for slice timing and rearranged. (2) The functional image and T1 image were repositioned with six rigid body parameters and registered in the functional space together with segmentation. (3) The structural image was segmented uniformly and normalized. (4) White matter and cerebrospinal fluid signals were regressed with the Friston 24-parameter model, and the head motion was corrected again. (5) The image was resampled into a 3-mm cube element and then smoothed with a Gaussian kernel with a half-maximum width of 6 mm. (6) The imaging data were time filtered (0.01–0.08 Hz) to eliminate the effects of extremely low-frequency drift and high-frequency noise (such as respiration and heart rhythm) and generate fALFF map. (7) Conversion of an fALFF map to a zfALFF map using a normal *Z* transform.

#### Clinical Data and Correlation Analysis

Paired *t*-tests (normal distribution) or Wilcoxon signed rank tests (non-normal distribution) were selected for within-group comparisons. Two simple *t*-tests (normal distribution) or Mann–Whitney *U*-test (non-normal distribution) was chosen for between-group comparisons. The level of statistical significance was *p* < 0.05, and a two-tailed test was used. Correlation analysis was performed using Pearson correlation analysis (normal distribution) or Spearman correlation analysis (non-normal distribution). The level of statistical significance was *p* < 0.05, and a two-tailed test was used.

#### Analysis of Effect-Related Brain Regions

In the first step, the paired *t*-test was used to analyze the changed brain areas in 115 FD patients after acupuncture treatment and to extract the change values (*p* < 0.05, GRF-corrected) (Liu et al., 2021). In the second step, clinical improvement values were calculated for 115 FD patients after acupuncture treatment. In the third step, a correlation analysis of the results of the first and second steps was performed to obtain the brain areas associated with the efficacy of acupuncture.

#### Grouping of Higher or Lower Response to Acupuncture

The 115 FD patients were regrouped according to the efficacy. Referring to the study by Ma et al. (2015), patients with FD were divided into two subgroups based on a median SID improvement value of 2 points: the higher response group (greater than or equal to 2 points) and the lower response group (less than 2 points).

#### Resting-State Functional Connection Analysis

Paired *t*-test was used to analyze the brain activities of patients in the acupuncture higher/lower response group, and the brain regions related to efficacy in the higher response group were regarded as regions of interest (ROI). The paired *t*-test was used to analyze the resting-state functional connectivity (rsFC) before and after the acupuncture treatment in the higher response group. This study selected all comparison thresholds for brain regions with GRF-corrected *p* < 0.05 (Liu et al., 2021).

## Results

### Cerebral Activity Changes and the Correlation With Symptom Improvement in All Functional Dyspepsia Patients

#### Cerebral Activity Changes Elucidated by Acupuncture Treatment in All Functional Dyspepsia Patients

**FIGURE 1:**
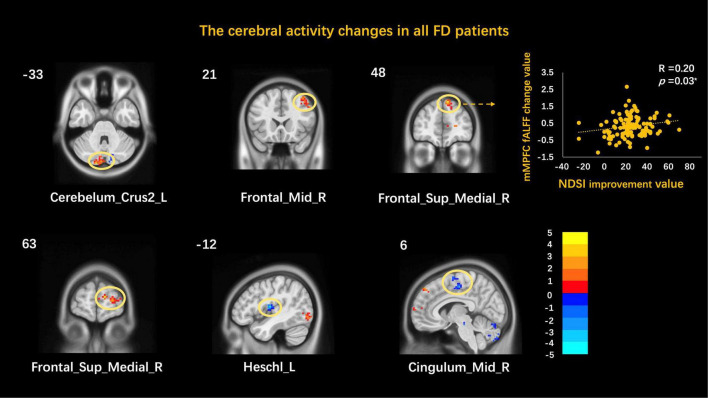
Correlation between cerebral activity changes and symptom improvement. Cerebelum_Crus2, cerebellum inferior; Frontal_Mid, middle temporal gyrus; Frontal_Sup_Medial, medial prefrontal cortex; Cingulum_Mid, middle cingulate cortex; L, left; R, right; *p < 0.05.

The functional activities of the left cerebellum inferior, right middle temporal gyrus, and right medial prefrontal cortex increased while those of the left Heschl and the right middle cingulate cortex decreased after acupuncture treatment for all FD patients (Table 1 and Figure 1).

**TABLE 1:** Cerebral activity changes elucidated by acupuncture treatment in all FD patients.

			MNI				Cluster	Correlation with NDSI
Regions	Side	Sign	X	Y	Z	T value	size	*p* value
Cerebelum_Crus2	L	↑	−15	−90	−33	3.62	57	0.34
Frontal_Mid	R	↑	39	21	51	3.30	54	0.55
Frontal_Sup_Medial	R	↑	9	48	48	4.01	59	0.03*
Frontal_Sup_Medial	R	↑	12	63	12	3.91	85	0.11
Heschl	L	↓	−45	−12	12	−4.80	51	0.23
Cingulum_Mid	R	↓	6	−15	51	−3.92	134	0.45

MNI, Montreal Neurological Institute; Cerebelum_Crus2, cerebellum inferior; Frontal_Mid, middle temporal gyrus; Frontal_Sup_Medial, medial prefrontal cortex; Cingulum_Mid, middle cingulate cortex; ↑, increasing; ↓, decreasing; L, left; R, right; *p < 0.05.

#### The Correlation Between Cerebral Activity Changes and Symptom Improvement

The correlation analysis showed that the change of the right medial prefrontal cortex (mPFC) was correlated with the improvement of NDSI (*r* = 0.20, *p* = 0.03) but not with the improvement in SID. Other brain function activity areas were not associated with the clinical improvement value (Table 1 and Figure 1).

### Cerebral Activity Changes in the Acupuncture Higher/Lower Response Group

#### Grouping Results of Demographic Characteristics and Clinical Variables

Among the 115 FD patients, 66 FD patients were assigned to the higher response group, and 49 FD patients were assigned to the lower response group. The demographic characteristics and clinical variables of the two groups are described in Table 2. There was no significant difference between gender, age, height, and weight between the two groups.

**TABLE 2:** Demographic characteristics and clinical variables in all FD patients/higher/lower groups.

	All FD patients (*n* = 115)	Higher response group (*n* = 66)	Lower response group (*n* = 49)	*p* value
Gender (male/female)	28/88	16/50	11/38	1
Age (years), mean ± SD	22.22 ± 2.34	22.00 ± 1.98	22.55 ± 2.70	0.47
Height, mean ± SD	161.93 ± 7.64	162.12 ± 7.00	161.71 ± 8.56	0.63
Weight, mean ± SD	52.52 ± 8.61	51.62 ± 8.91	53.85 ± 8.13	0.24
NDSI score improvements, mean ± SD				
	23.67 ± 15.68	29.36 ± 13.40	15.90 ± 15.34	0.00**
SID score improvements, median (IQR)				
	2 (1,3)	3 (2,4)	1 (0,1)	0.00**

NDSI, Nepean Dyspepsia Symptom Index; SID, Symptom Index of Dyspepsia; **p < 0.001.

#### Cerebral Activity Changes of the Higher/Lower Groups

**FIGURE 2:**
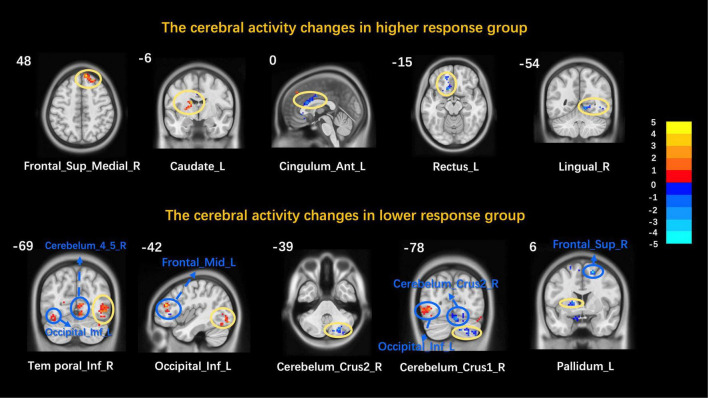
Cerebral activity changes in the higher/lower response group. Frontal_Sup_Medial, medial prefrontal cortex; Cingulum_Ant, anterior cingulate cortex; Lingual, lingual gyrus; Temporal_Inf, inferior temporal gyrus; Occipital_Inf, inferior occipital gyrus; Cerebelum_Crus2, cerebellum inferior; Cerebelum_Crus1, cerebellum superior; L, left; R, right. Yellow circles mark peak points and blue marks non-peak areas.

After acupuncture treatment, the functional activities of the right mPFC and the left caudate increased while those of the left anterior cingulate cortex, the left rectus, and the right lingual gyrus decreased in the higher response group. After acupuncture treatment, the functional activities of the right inferior temporal gyrus and the left inferior occipital gyrus increased while those of the right cerebellum inferior, the right cerebellum superior, and the left pallidus decreased in the lower response group. The two groups’ detailed information of altered brain areas after acupuncture treatment is described in Table 3 and Figure 2.

**TABLE 3:** The cerebral activity changes in higher/lower group.

			MNI				
Regions	Side	Sign	X	Y	Z	T value	Cluster size
**Higher response group**							
Frontal_Sup_Medial	R	↑	9	48	48	4.10	61
Caudate	L	↑	−15	−6	18	3.95	63
Cingulum_Ant	L	↓	0	18	24	−3.50	72
Rectus	L	↓	−12	21	−15	−3.30	70
Lingual	R	↓	12	−54	−6	−3.15	79
**Lower response group**							
Temporal_Inf	R	↑	48	−69	−6	3.87	73
Occipital_Inf	L	↑	−42	−84	−12	3.51	53
Cerebelum_Crus2	R	↓	42	−75	−39	−4.08	76
Cerebelum_Crus1	R	↓	6	−78	−21	−3.06	68
Pallidum	L	↓	−18	3	6	−3.40	71

*MNI, Montreal Neurological Institute; Frontal_Sup_Medial, medial prefrontal cortex; Cingulum_Ant, anterior cingulate cortex; Lingual, lingual gyrus; Temporal_Inf, inferior temporal gyrus; Occipital_Inf, inferior occipital gyrus; Cerebelum_Crus2, cerebellum inferior; Cerebelum_Crus1, cerebellum superior; ↑, increasing; ↓, decreasing; L, left; R, right.*

### Resting-State Cerebral Functional Connectivity in the Higher Response Group

**FIGURE 3:**
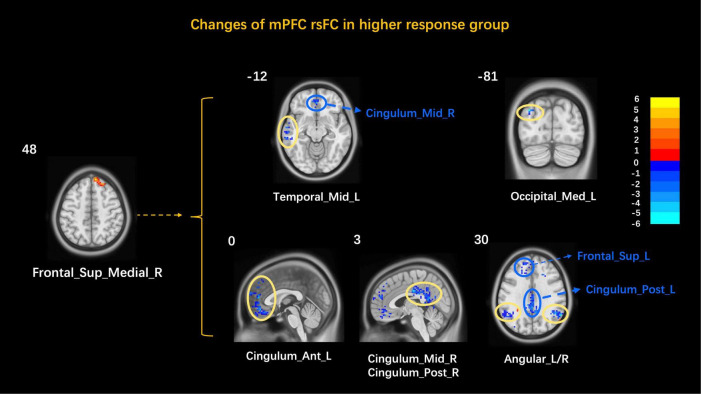
Changes of the mPFC rsFC in the higher response group. Frontal_Sup_Medial, medial prefrontal cortex; Temporal_Mid, middle temporal gyrus; Cerebelum_Crus1, cerebellum superior; Cingulum_Ant, anterior cingulate cortex; Cingulum_Mid, middle cingulate cortex; Cingulum_Post, posterior cingulate cortex; L, left; R, right. Yellow circles mark peak points and blue marks non-peak areas.

After finding that mPFC correlated with clinical improvement values and significant changes in functional activity in the higher response group, the mPFC was used as an ROI to explore the brain network characteristics of FD patients in the higher response group to acupuncture. The results showed that the mPFC rsFC with the left middle temporal gyrus, the right cerebellum superior, the left anterior cingulate cortex, the right middle cingulate cortex/posterior cingulate cortex, and the bilateral angular gyrus decreased (Table 4 and Figure 3). The correlation analysis showed no correlation between the mPFC rsFC and clinical symptom improvement values in the higher response group.

**TABLE 4:** Changes of mPFC rsFC in higher response group.

			MNI				
Regions	Side	Sign	X	Y	Z	T value	Cluster size
Temporal_Mid	L	↓	−63	−30	12	−4.01	176
Cerebelum_Crus1	R	↓	45	−81	−33	−3.75	103
Cingulum_Ant	L	↓	0	45	12	−4.45	409
Cingulum_Mid/Cingulum_Post	R	↓	3	−18	33	−5.03	379
Angular	L/R	↓	60	−57	30	−4.79	187

*MNI, Montreal Neurological Institute; Temporal_Mid, middle temporal gyrus; Cerebelum_Crus1, cerebellum superior; Cingulum_Ant, anterior cingulate cortex; Cingulum_Mid, middle cingulate cortex; Cingulum_Post, posterior cingulate cortex; ↓, decreasing; L, left; R, right.*

### Resting-State Cerebral Functional Connectivity in the Lower Response Group

**FIGURE 4:**
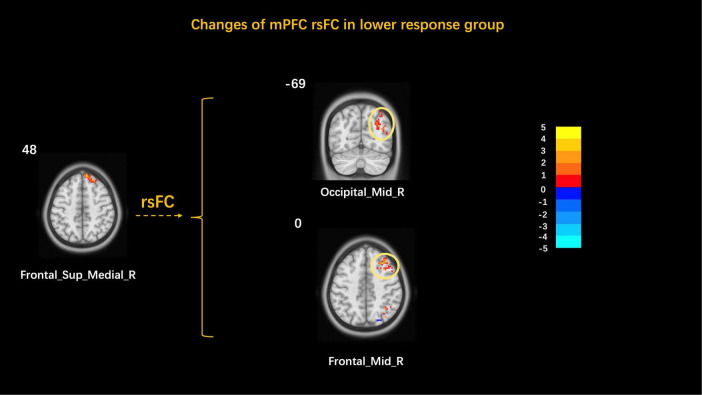
Changes of the mPFC rsFC in the higher response group. Occipital_Mid, middle occipital gyrus; Frontal_Mid_Orb, orbital middle frontal gyrus; L, left; R, right.

The mPFC was used as the ROI to explore the brain network characteristics of FD patients in the acupuncture low response group. The results showed that the mPFC rsFC with the right middle occipital gyrus and the right orbital middle frontal gyrus increased (Table 5 and Figure 4). The correlation analysis showed no correlation between the mPFC rsFC and clinical symptom improvement values in the lower response group.

**TABLE 5:** Changes of mPFC rsFC in the lower response group.

			MNI				
Regions	Side	Sign	X	Y	Z	T value	Cluster size
Occipital_Mid	R	↑	33	−69	30	4.09	151
Frontal_Mid_Orb	R	↑	30	45	0	3.995	78

*MNI, Montreal Neurological Institute; Occipital_Mid, middle occipital gyrus; Frontal_Mid_Orb, orbital middle frontal gyrus; ↑, increasing; ↓, decreasing; L, left; R, right.*

### Between-Group Comparisons of Resting-State Cerebral Activity Changes in the Higher and Lower Response Groups

**FIGURE 5:**
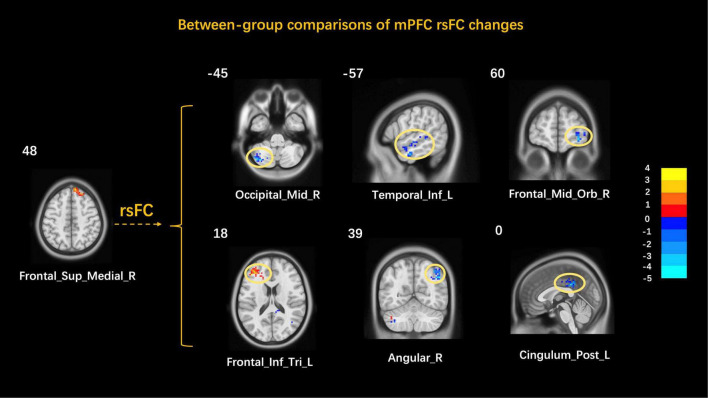
Between-group comparisons of mPFC rsFC changes. MNI, Montreal Neurological Institute; Cerebelum_Crus2, cerebellum inferior; Temporal_Inf, inferior temporal gyrus; Frontal_Mid_Orb, orbital middle frontal gyrus; Frontal_Inf_Tri, inferior frontal gyrus, triangular part; Cingulum_Post, posterior cingulate cortex; L, left; R, right.

A comparison of the mPFC rsFC in the higher response and lower response groups was done to explore the brain network characteristics of FD patients with the acupuncture higher/lower response groups. The results showed that the mPFC rsFC with the left cerebellum inferior, the left inferior temporal gyrus, the right orbital middle frontal gyrus, the right angular gyrus, and the left posterior cingulate cortex decreased while that with the left inferior frontal gyrus triangular part increased (Table 6 and Figure 5).

**TABLE 6:** Between-group comparisons of mPFC rsFC changes.

			MNI				
Regions	Side	Sign	X	Y	Z	T value	Cluster size
Cerebelum_Crus2	L	↓	−36	−72	−45	−4.09	94
Temporal_Inf	L	↓	−57	−9	−27	−3.67	93
Frontal_Mid_Orb	R	↓	30	60	−9	−3.79	101
Frontal_Inf_Tri	L	↑	−42	36	18	3.76	124
Angular	R	↓	48	−60	39	−4.45	221
Cingulum_Post	L	↓	0	−36	33	−4.12	96

*MNI, Montreal Neurological Institute; Cerebelum_Crus2, cerebellum inferior; Temporal_Inf, inferior temporal gyrus; Frontal_Mid_Orb, orbital middle frontal gyrus; Frontal_Inf_Tri, inferior frontal gyrus, triangular part; Cingulum_Post, posterior cingulate cortex; ↑, increasing; ↓, decreasing; L, left; R, right.*

## Discussion

By analyzing the correlation between cerebral activity changes and symptom improvements of all FD patients after acupuncture treatment and comparing the differences in cerebral activity changes between the higher response and the lower response groups, this study found that mPFC might be involved in acupuncture for FD and demonstrated the alternations of functional connectivity between mPFC and default mode network (DMN).

Several studies have confirmed that the mPFC is involved in regulating gastrointestinal physiological function and in the central pathological changes of FD (Kano et al., 2020). In a physiological state, the mPFC is extensively involved in the cognitive process, visceral function regulation, emotional activity, etc. (Euston et al., 2012). The mPFC includes the infralimbic cortex (IL) and the prelimbic cortex (PL) (Hurley-Gius and Neafsey, 1986; Neafsey, 1990). The former exerts a pronounced influence on visceral/autonomic nervous activities through its direct projections to the medulla gastrointestinal motor centers. At the same time, some cortical neurons that affect the output of the gastric parasympathetic nerve originate from the mPFC, so IL is considered to belong to the “visceral motor cortex.” PL is related to cognitive, emotional, and executive functions and is considered part of the “cognitive–emotional cortex” (Zhao et al., 2019). In a pathological state, the structural and functional changes in the mPFC in FD patients have been documented in multiple neuroimaging studies. For example, an 18F-FDG PET-CT study found that FD patients showed significant glucometabolic increase in mPFC, which suggests that the altered signal of mPFC might be related to patients’ selective attention to sensations from the stomach, such as postprandial upper abdominal discomfort, early satiety, and abdominal distension (Zeng et al., 2011). A structural MRI study reported the changes of cortical thickness and subcortical volume in mPFC in FD patients compared with healthy subjects and suggested that these changes were closely related to the severity of symptoms (Liu et al., 2018). Similarly, a decreased gray matter density of mPFC was found in FD patients reporting meal-related symptoms compared with healthy subjects (Zeng et al., 2013). These studies demonstrated the role of mPFC in the central pathogenesis of FD. They suggested that mPFC may be a potential target brain region of gastrointestinal modulating effect of acupuncture treatment.

To investigate the role of mPFC in the acupuncture treatment for FD, three analyses were designed in this current study. Firstly, significant cerebral activity changes in mPFC were found in all FD patients after 4 weeks of acupuncture treatment. The increased activity of mPFC was markable related to the improvement in NDSI scores. It indicated that the more significant the decrease in mPFC activity, the more influential the dyspepsia symptoms. The results suggested that the regulation of mPFC function may be one of the mechanisms of acupuncture for treating FD. Secondly, to further confirm the involvement of mPFC in the therapeutic effect of acupuncture on FD, 115 FD patients included in this study were divided into the higher response group and the lower response group according to the clinical improvement of SID scores. The results showed increased activities in the mPFC of the higher response FD patients, while there were no significant activities in the mPFC of the lower response patients. These two results indicate that mPFC should be a critical region in the realization of acupuncture effect, and the adjustment of abnormal function of mPFC may be an important mechanism of acupuncture effectively treating FD. Thirdly, to further explore how mPFC participates in the realization of acupuncture effect, this study selected the mPFC as ROI and performed the ROI-based rsFC analysis. The results demonstrated that the rsFC of the mPFC with the left anterior cingulate cortex (ACC), the right middle cingulate cortex (MCC)/posterior cingulate cortex (PCC), and the bilateral angular gyrus (AG) significantly decreased in the higher response group but not in the lower response group. Also, the results of the between-group comparison between the high and low response groups showed that the functional connectivity of mPFC rsFC with regions such as PCC and AG was decreased. These decreased regions are an important part of the DMN.

The DMN is a functional network composed of brain regions that maintain synchronous, low-frequency spontaneous neuronal activity in a resting state and continue to be negatively activated in a task state (Lei et al., 2013) and is thought to be associated with social behavior, emotional control, and sensory-visceral movements. Several neuroimaging studies have demonstrated the DMN alternations in patients with gastrointestinal diseases, including FD (Liu et al., 2013), functional constipation (FC) (Yin et al., 2021), irritable bowel syndrome (IBS) (Qi et al., 2016), Crohn’s disease (CD) (Thomann et al., 2017), and ulcerative colitis (UC) (Skrobisz et al., 2020), and suggested the involvement of DMN in the abnormal process of brain–gut interaction in these gastrointestinal diseases. For example, a structural MRI indicated that, compared with healthy subjects, FD patients showed significant cortical thickness alterations in some regions of DMN such as mPFC, PCC, AG, etc. (Liu et al., 2018). Furthermore, a review indicated that functional connectivity changes in DMN were commonly seen in acupuncture studies with different participants. The involvement of DMN in acupuncture treatment is suggested to be a factor in the realization of treatment efficacy. For example, previous studies found that acupuncture could enhance the post-stimulus spatial extent of DMN in healthy subjects (Dhond et al., 2008) and could modulate the abnormal function of DMN in IBS patients (Zhao et al., 2018) and so on. In this study, the functional connectivity between mPFC and DMN was significantly decreased in the higher response group after acupuncture treatment. The results suggested that the efficacy of acupuncture for FD may be achieved by modulating the internal connectivity pattern between the mPFC and the DMN to improve the abnormal gastric sensory and cognitive/emotional processing. The limitations of this study are mainly that the results of this study are a secondary analysis of two randomized controlled neuroimaging studies of acupuncture for FD, and the results need to be verified in a subsequent strictly designed prospective study.

## Conclusion

In conclusion, this study discovered that mPFC might play an important role in the acupuncture treatment for FD from three aspects. Based on finding the significantly decreased functional activity of mPFC and its negative correlation with symptom improvement in all FD patients after acupuncture treatment, a subgroup analysis further verified the participation of mPFC in acupuncture treatment efficacy, and a ROI-based functional connectivity analysis indicated the enhancement in functional connectivity between mPFC and DMN in the higher response group. These results suggest that modulating the functional activity of the mPFC and its connectivity to the DMN may be one of the important mechanisms of acupuncture for treating FD. This implies that mPFC and DMN might play a key role in the treatments for FD by acupuncture.

## Data Availability Statement

The original contributions presented in the study are included in the article/supplementary material, further inquiries can be directed to the corresponding author/s.

## Ethics Statement

The studies involving human participants were reviewed and approved by Affiliated Hospital of Chengdu University of Traditional Chinese Medicine. The ethics committee waived the requirement of written informed consent for participation. Written informed consent was obtained from the individual(s) for the publication of any potentially identifiable images or data included in this article.

## Author Contributions

FZ, YT, and YY designed the study and drafted the manuscript. FZ, YC, and TY revised the study design and the manuscript. TY, RS, PM, ZH, YQ, and LH were involved in the study implementation and data collection. ZT was involved in the design of the diagrams in the manuscript. All authors contributed to the article and approved the submitted version.

## Conflict of Interest

The authors declare that the research was conducted in the absence of any commercial or financial relationships that could be construed as a potential conflict of interest.

## Publisher’s Note

All claims expressed in this article are solely those of the authors and do not necessarily represent those of their affiliated organizations, or those of the publisher, the editors and the reviewers. Any product that may be evaluated in this article, or claim that may be made by its manufacturer, is not guaranteed or endorsed by the publisher.

## Footnotes

1
http://www.fil.ion.ucl.ac.uk/spm

2
http://rfmri.org/DPARSF
